# Impact of nutritional support on mortality among critically ill patients with different nutritional risks: a systematic review with meta-analysis

**DOI:** 10.3389/fnut.2025.1667389

**Published:** 2025-11-18

**Authors:** Lingling Bao, Youquan Wang, Yuting Li, Deyou Zhang, Hongxiang Li

**Affiliations:** Department of Critical Care Medicine, The First Hospital of Jilin University, Changchun, China

**Keywords:** nutritional support, critically ill patients, mNUTRIC score, energy intake, protein intake

## Abstract

**Background:**

To identify appropriate nutritional support strategies for critically ill patients with different levels of nutritional risk.

**Methods:**

A systematic search of PubMed, MEDLINE, Cochrane Library, and Embase was conducted from database inception to 19 May 2025, which included critically ill patients classified into high risk (5–9) and low risk (0–4) groups based on the modified Nutrition Risk in the Critically Ill (mNUTRIC) score. Data on study characteristics, patient demographics, and nutritional support details were extracted. The primary outcome was all-cause mortality following nutritional support stratified by nutritional risk among critically ill patients. A meta-regression analysis was performed to assess the influence of covariates on effect sizes and to identify potential sources of heterogeneity. Trial sequential analysis (TSA) was conducted to evaluate the robustness and reliability of the pooled effect estimates.

**Results:**

Eleven eligible trials, comprising a total of 7,442 participants, were included in this systematic review. The meta-analysis demonstrated that high nutritional risk was significantly associated with increased mortality (OR: 2.26, 95% CI: 1.80–2.83, *p* < 0.0001). Adequate energy intake was associated with a significantly lower 28-day mortality among patients at high nutritional risk (OR: 0.60, 95% CI: 0.38–0.94, *p* = 0.03). However, in randomized controlled trials, adequate energy support did not reduce 28-day mortality (OR: 1.09, 95% CI: 0.74–1.60) or 90-day mortality (OR: 1.03, 95% CI: 0.87–1.23) in high-risk patients.

**Conclusion:**

The mNUTRIC score is a validated prognostic tool in critically ill patients, but its effectiveness in guiding energy support remains limited.

**Systematic Review registration:**

https://www.crd.york.ac.uk/PROSPERO/view/CRD42020188064, Identifier: CRD42020188064.

## 1 Introduction

Critically ill patients who remain in the intensive care units (ICU) for more than 48 h are at a high risk of malnutrition (1), which is independently associated with poor outcomes such as prolonged ICU and hospitalization, prolonged mechanical ventilation, and higher incidences of infectious complications (2). Therefore, optimizing nutritional support during ICU care is essential for enhancing long term outcomes and reducing the risk of patients becoming adversely affected by critical illness (3, 4).

Prospective observational studies on nutrition practices in ICU worldwide have demonstrated that sufficient nutritional support can improve patient prognosis (5–8). However, several other studies have indicated that nutritional support provided during the acute phase had minimal impact on clinical outcomes and may even be harmful to critically ill patients (9–13). One possible explanation for these inconsistency among findings is the heterogeneity among patients from different studies (5). Heyland et al. (14) developed the Nutrition Risk in the Critically Ill (NUTRIC) score, which incorporates the severity of illness into its calculation and aims to quantify the risk of malnutrition and identify patients who would benefit from adequate nutritional support. Since quantifying interleukin-6 in typical clinical settings is difficult, a modified NUTRIC (mNUTRIC) score has been devised to eliminate interleukin-6 levels while preserving other relevant clinical indicators (15).

Previous research suggests that the mNUTRIC score might serve as an indicator for determining the need for intensive nutritional support in these patients (16–18) and as a predictor of mortality (19–21). However, it is unclear whether patients with higher nutritional risk actually derive greater survival benefit from adequate energy and protein intake.

The objective of this meta-analysis is to determine whether adequate energy and protein nutritional support is associated with reduced mortality in critically ill patients stratified by nutritional risk and to evaluate the prognostic value of the mNUTRIC score in this context.

## 2 Methods

### 2.1 Search strategy

This systematic review and meta-analysis was conducted following the Preferred Reporting Items for Systematic Reviews and Meta-Analysis (PRISMA) guidelines. The study protocol was registered with PROSPERO database (CRD42020188064). Two reviewers conducted independent searches of PubMed, MEDLINE, Cochrane Library, and Embase databases from their inception to 19 May 2025. The search included the terms ‘nutritional support,’ ‘nutrition therapy,’ ‘nutritional risk,’ ‘NUTRIC score,’ ‘mNUTRIC score,’ ‘critical care,’ ‘intensive care,’ and ‘critically ill.’ Only articles published in English were included.

### 2.2 Study selection

Eligible studies included randomized controlled trials (RCTs), cohort studies, post-hoc analysis, and both prospective and retrospective observational studies. Research focusing on critically ill patients, specifically those categorized by the mNUTRIC scores, were prioritized for inclusion. Patients were categorized into high risk (5–9) and low risk (0–4) groups based on their mNUTRIC score.

### 2.3 Inclusion and exclusion criteria

The inclusion criteria were as follows: (1) clinical trials; (2) studies involving critically ill patients classified according to the mNUTRIC score; (3) studies evaluating nutritional support administered through enteral and/or parenteral nutrition; (4) comparisons between patients receiving adequate versus inadequate energy and/or protein intake; and (5) studies reporting mortality as an outcome measure. The primary outcome was overall mortality, including 28-day, 30-day, 60-day, and 90-day mortality. The exclusion criteria were as follows: (1) studies that did not report or classify patients according to the mNUTRIC score; (2) studies lacking a clear definition or comparison of adequate versus inadequate energy and/or protein intake; (3) studies with insufficient data to extract or calculate effect estimates; (4) studies enrolling non-critically ill populations or employing other nutritional risk tools instead of the mNUTRIC score; and (5) reviews, editorials, case reports, conference abstracts, or non-English publications.

### 2.4 Data extraction

Two reviewers independently extracted data from all eligible studies using a predefined spreadsheet. Extracted variables included study design, inclusion criteria, baseline patient characteristics, definitions of adequate nutritional support, and mortality outcomes. Discrepancies were addressed via consensus.

### 2.5 Quality assessment

The risk of bias in RCTs was assessed using the Cochrane Risk of Bias Tool, which evaluates domains including selection, performance, detection, attrition, and reporting biases. The Newcastle-Ottawa Scale was utilized for observational studies, emphasizing selection, comparability, and outcome assessment. Due to the limited number of included studies, the calculation of inter-rater reliability (Cohen’s *κ*) would be statistically unstable and was not performed. Any discrepancies between the two reviewers were resolved through discussion.

### 2.6 Definition of adequate energy and protein intake

Adequate nutritional support was defined according to the criteria used in the included studies. Specifically, adequate energy intake referred to 65–100% of the prescribed energy target, while adequate protein intake was defined as 1.0–1.5 g/kg per day or at least two-thirds of the prescribed protein target.

### 2.7 Statistical analysis

All statistical analyses were performed using R software (version 4.2.2, R Foundation for Statistical Computing, Vienna, Austria). The primary outcome was mortality, which were statistically represented by odds ratios (ORs) with 95% confidence intervals (CIs). A random-effects model was used for studies with significant heterogeneity (*p* < 0.10, I^2^ > 50%).

A leave-one-out sensitivity analysis was performed using the metainf function from the meta package in R. Each study was sequentially excluded from the meta-analysis to assess its influence on the overall odds ratio (OR), and the robustness of the pooled effect was evaluated accordingly.

Trial sequential analysis (TSA) was conducted using TSA software (v0.9.5.10, Copenhagen Trial Unit) with a two-sided *α* of 5 and 80% statistical power. The required information size (RIS) was estimated assuming a 24% relative risk reduction for 28-day mortality and 22.5% reduction for 90-day mortality, applying the O’Brien-Fleming *α*-spending function and variance-based heterogeneity correction under a random-effects model. TSA was performed to control the risk of random errors due to repeated significance testing and to assess whether the cumulative evidence was sufficient to support firm and reliable conclusions.

## 3 Result

### 3.1 Study characteristics and quality assessment

**Figure 1:**
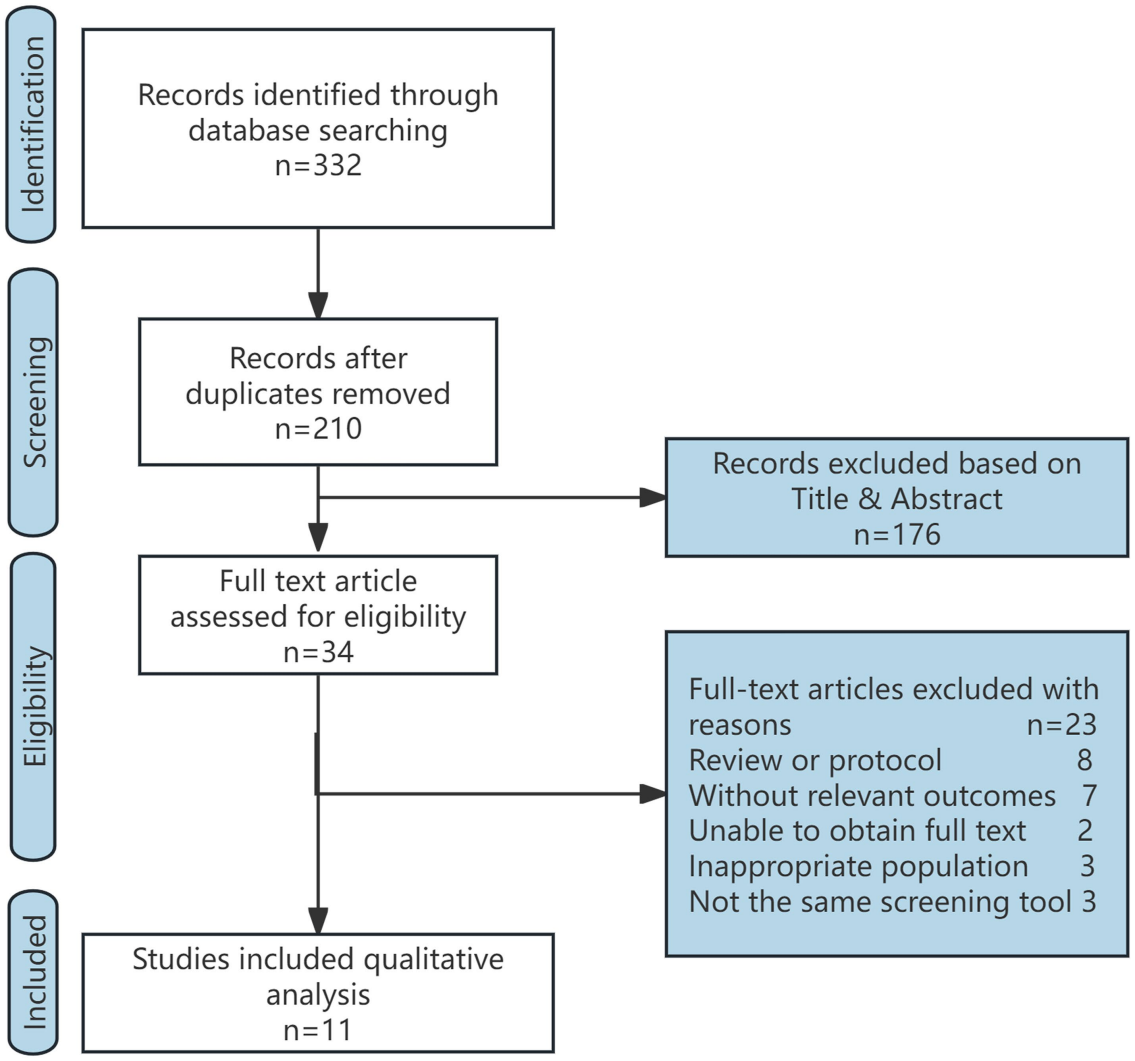
Study selection flow diagram.

A total of 332 articles were initially identified. After screening the titles and abstracts, 34 studies were deemed potentially eligible. The full text articles of these 34 studies were reviewed individually, and 11 studies (22–32) were deemed eligible for further analysis. Study Selection Flow Diagram is shown in Figure 1. Among the included studies, 11 studies focused on energy administration, of which 4 (22, 25, 27, 28) investigated both energy and protein intake. The 11 eligible trials included a total of 7,442 participants, and the key characteristics of these studies are summarized in Table 1.

**Table 1:** Characteristics of included studies.

Authors (years)	Study period	Country or Region	Study design	Inclusion criteria	Total number of patients (high risk vs. low risk)	Age (high risk vs. low risk)	APACHE II score (high risk vs. low risk)	BMI (kg/m^2^) (high risk vs. low risk)	SOFA	Route of nutrition support	Mortality	Investigated duration of nutrition support	Definition of energy adequacy	Definition of protein adequacy (if applicable)	Target energy requirement	Target protein requirement (if applicable)
Arabi (2017)(23)	2009–2014	Saudi Arabia and Canada	Post-hoc Analysis of Multicenter RCT	Critically ill patients	894 (378vs 513)	61.5 ± 15.6 vs. 42.5 ± 17.98	26.5 ± 6.67 vs. 17 ± 5.54	28.2 ± 8.08 vs. 27.2 ± 6.43	11.7 ± 3.04 vs. 8.5 ± 3.15	EN	28-day mortality	The first 14 days in the ICU	Exceeding 70% of energy requirement	NA	25-30 kcal/kg/day	1.2–1.5 g/kg/day
Lee (2018)(22)	2015–2016	Malaysia	Prospective Observational Study	Critically ill patients with MV	154 (86 vs. 68)	51.3 ± 15.73*	28.9 ± 7.35*	26.52 ± 6.65*	12.4 ± 3.66*	EN + PN	60-day mortality	The first 12 days in the ICU	Exceeding 66.7% of energy requirement	Exceeding 66.7% of protein requirement	25 kcal/kg/day	1.2 g/kg/day
Hung (2019) (26)	2013–2016	Taiwan	Prospective Observational Study	Critically ill patients with sepsis	122 (71 vs. 51)	69.6 ± 15.6*	26 ± 8.77 *	22.8 ± 4.7*	10.7 ± 4.6*	EN	28-day mortality	The first 7 days in the ICU	Exceeding 65% of energy requirement	NA	25–30 kcal/kg/day	1.2–1.5 g/kg/day
Jeong (2019) (27)	2011–2017	Republic of Korea	Retrospective Cohort Study	Critically ill patient with sepsis	248 (220 vs. 28)	65 ± 12.59 vs. 54 ± 20.74	24 ± 5.92 vs. 15 ± 3.7	23 ± 4.44 vs. 21 ± 4.44	12 ± 3.7 vs. 6 ± 3.0	EN + PN	28-day mortality	The first 7 days in the ICU	Exceeding 80% of energy requirement	Exceeding 1.0 g/kg/day	25–30 kcal/kg/day	1.2–1.5 g/kg/day
Wang (2020) (31)	2017–2020	Taiwan	RCT	Critically ill patients	150 (106 vs. 44)	71.2 ± 13.5 vs. 58.3 ± 19	28.3 ± 4.78 vs. 19.8 ± 4.24	23.8 ± 4.89 vs. 23.7 ± 6.15	NA	EN + PN	28-day mortality	The first 6 days in the ICU	Exceeding 100% of energy requirement	NA	25 kcal/kg/day	1.2 g/kg/day
Chada (2021) (24)	2019	India	Prospective Observational Study	Critically ill patients	248 (95 vs. 153)	64.9 ± 12.7 vs. 58.3 ± 15.3	23 ± 5.8 vs. 13 ± 4.7	23.9 ± 4.9 vs. 24.3 ± 4.7	8.3 ± 3.22 vs. 5.6 ± 3.15	EN	28-day mortality	The first 5 days in the ICU	Exceeding 80% of energy requirement	NA	25–30 kcal/kg/day	1.2–1.5 g/kg/day
Jung et al. (2018)(32)	2007–2017	Republic of Korea	Retrospective cohort study	Surgical and septic patient with MV in ICU	215 (108 vs. 107)	69.74 ± 11.45 vs. 53.36 ± 15.57	29.42 ± 5.77 vs. 20.88 ± 7.0	21.0 ± 3.8 vs. 22.0 ± 3.8	6.7 ± 3.1 vs. 4.6 ± 2.6	EN + PN	30-day mortality	The first 5 days in the ICU	Exceeding 70% of energy requirement	NA	25 kcal/kg/day	NA
Sim et al. (2021)(30)	2013–2018	South Korea	Retrospective Cohort Study	Critically ill patients after surgery	317 (111 vs. 206)	76.8 ± 15.45 vs. 66.5 ± 11.54	16.3 ± 9.48 vs. 11 ± 9.02	21.1 ± 3.97 vs. 22.6 ± 2.52	NA	PN	30-day mortality	The first 7 days in the ICU	Exceeding 80% of energy requirement	NA	25–30 kcal/kg/day	1.2–1.5 g/kg/day
Im and Kim (2023) (28)	2019–2022	South Korea	RCT	Critically ill patients after surgery	325 (108 vs. 217)	70.6 ± 12.8*	16.7 ± 6.7*	23 ± 4.5*	8.1 ± 3.3*	EN + PN	60-day mortality	NA	Exceeding 80% of energy requirement	Exceeding 1.2 g/kg/day	25–30 kcal/kg/day	1.2–1.5 g/kg/day
Hung (2023) (25)	2020–2022	Taiwan	RCT	Critically ill patients with sepsis	132 (93 vs. 39)	74.1 ± 11.5 vs. 63.7 ± 10	24.7 ± 5.7 vs. 14.8 ± 4.7	23.4 ± 5.6 vs. 22.9 ± 5.5	8 ± 4 vs. 5 ± 4	EN + PN	28-day mortality	The first 7 days in the ICU	Exceeding 80% of energy requirement	Exceeding 1.2 g/kg/day	25 kcal/kg/day	1.2 g/kg/day
Casaer (2024) (29)	2007–2011	Belgium	Prospective Follow-up Study of RCT	Critically ill patients	4,640 (2,829 vs. 1811)	66.3 ± 14.1*	19 ± 13.3*	26.01 ± 4.34*	NA	EN + PN	100-day mortality	The first 7 days in the ICU	Exceeding 100% of energy requirement	NA	25–30 kcal/kg/day	1.2–1.5 g/kg/day

ICU, Intensive Care Unit; APACHE II, Acute Physiology and Chronic Health Evaluation; BMI, Body Mass Index; SOFA, Sequential Organ Failure Assessment; EN, Enteral Nutrition; PN, Parenteral Nutrition; MV, Mechanical Ventilation; RCT, Randomized Controlled Trial; NA, Not Applicable. *The data were not stratified by nutritional risk level.

### 3.2 Quality assessment

The observational studies, assessed using the Newcastle–Ottawa Scale, were found to have a moderate risk of bias, with all studies rated as moderate quality (5*). The RCTs were evaluated using the Cochrane Risk of Bias tool and also showed a moderate risk of bias, primarily due to concerns related to the blinding of participants, personnel, and outcome assessment (Supplementary Table S1; Supplementary Figure S1).

### 3.3 Mortality associated with different nutrition risk in critically ill patients

**Figure 2:**
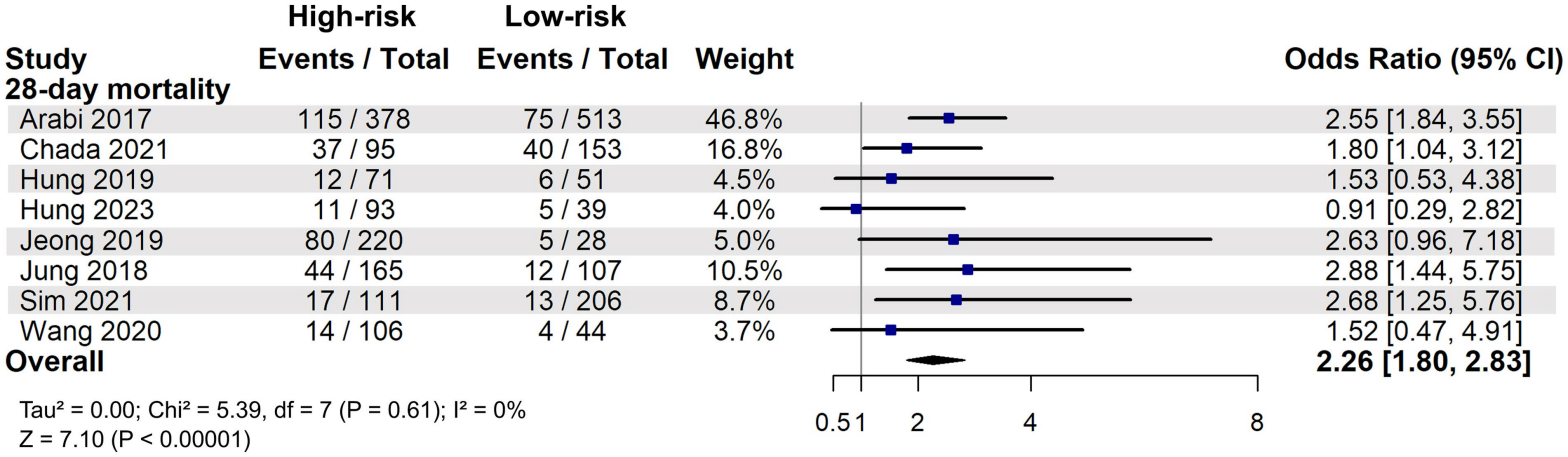
28-day mortality associated with different nutrition risk in critically ill patients.

To compare mortality between patients with high and low nutritional risk, a meta-analysis was performed by pooling data from studies that reported mortality outcomes stratified by nutritional risk (22–28, 30–32). The results demonstrated that high nutritional risk was significantly associated with increased 28-day mortality (OR: 2.26, 95% CI: 1.80–2.83, *p* < 0.00001, Figure 2). Sensitivity analyses, conducted by excluding each study in turn, showed consistent results, indicating the robustness of the findings (Supplementary Figure S2).

### 3.4 Mortality associated with energy adequacy in critically ill patients

**Figure 3:**
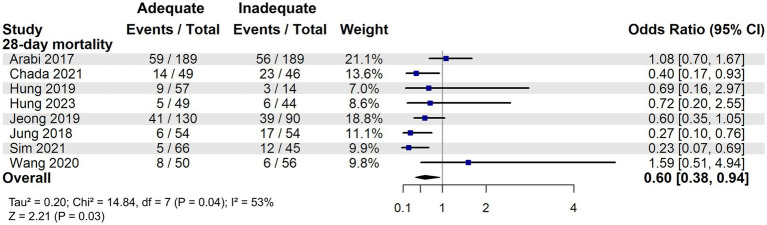
28-day mortality associated with energy adequacy in critically ill patients with high nutritional risk.

A total of 4,205 patients were identified as high nutritional risk patients based on the mNUTRIC score. The meta-analysis suggested that adequate energy intake might be associated with a reduction in 28-day mortality among patients at high nutritional risk (OR: 0.60, 95% CI: 0.38–0.94, *p* = 0.03) and that moderate heterogeneity was observed among the included studies (I^2^ = 53%, Figure 3).

**Figure 4:**
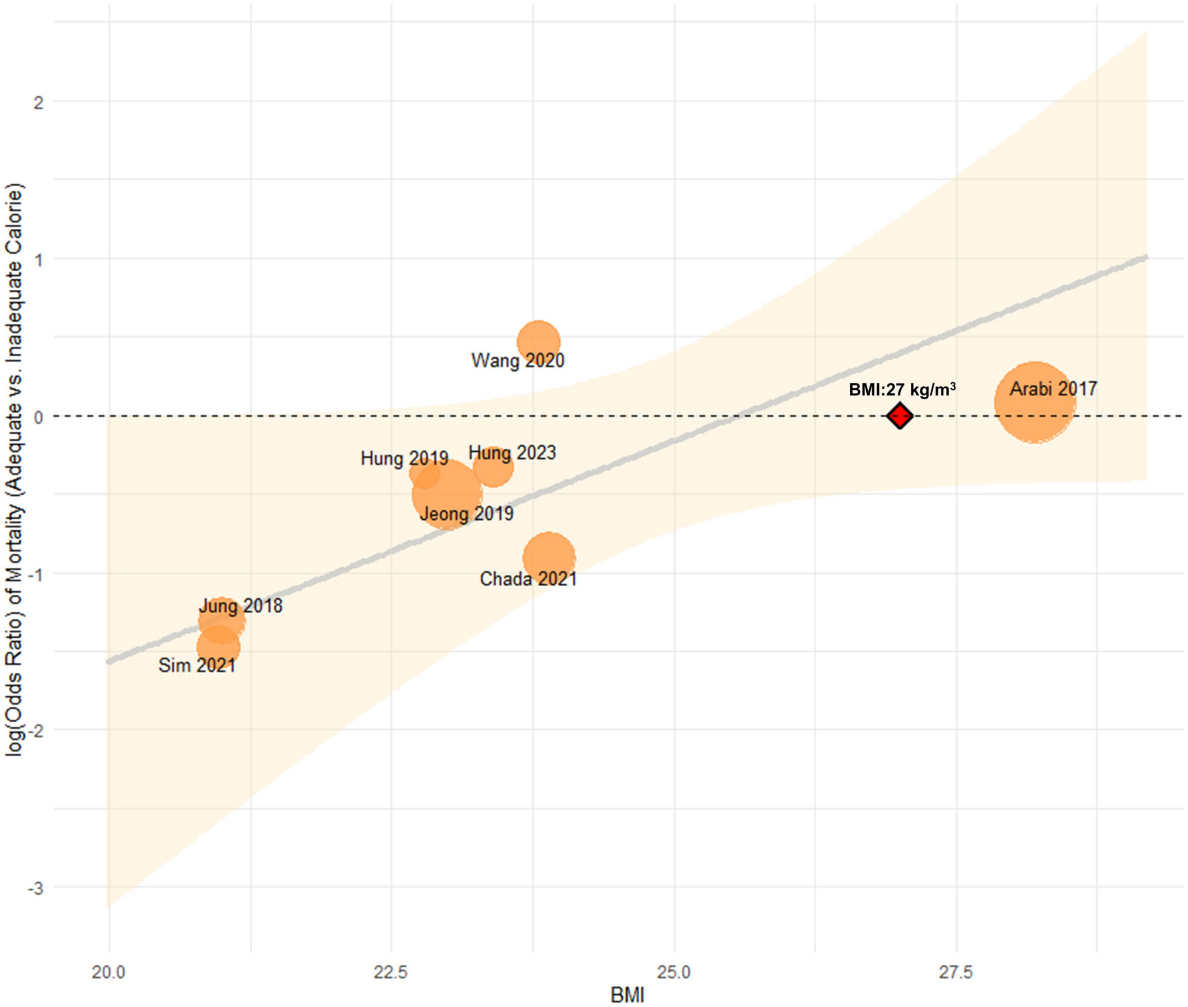
Association between BMI and the effect of energy intake on 28-day mortality in high nutritional risk.

To further explore heterogeneity, a multivariate metaregression analysis was performed in high nutritional risk patients. BMI and APACHE II score were initially identified as covariates potentially associated with the effect size (*p* < 0.1) and were included in the final model. This model significantly explained the heterogeneity (*R*^2^ = 100%, I^2^ = 0%; *p* = 0.0066). Among these, only BMI remained a significant moderator (*β* = 0.13, 95% CI: 0.02–0.24; *p* = 0.02). A potential interaction between BMI and the effect of adequate energy intake was also observed: patients with BMI < 27 kg/m^2^ appeared to benefit from adequate energy support, whereas those with BMI ≥ 27 kg/m^2^ showed no clear 28-day survival advantage (Figure 4). In addition, the meta-analysis of the effect of adequate energy support on 60-day mortality among high-risk patients is shown in Supplementary Figure S3.

**Figure 5:**
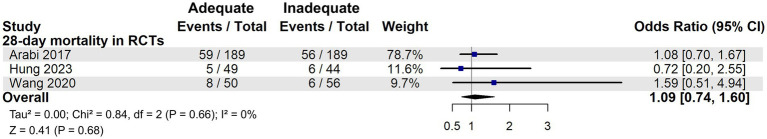
28-day mortality in critically ill patients with high nutritional risk: results from RCTs on energy adequacy.

**Figure 6:**
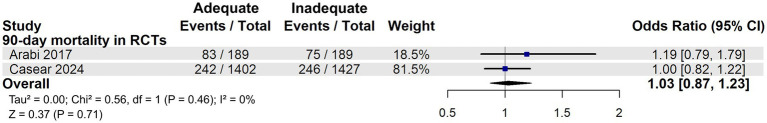
90-day mortality in critically ill patients with high nutritional risk: results from RCTs on energy adequacy.

However, when considering only RCTs, including two secondary analyses of RCTs [Arabi et al. (23) and Casaer et al. (29)], adequate energy support did not confer a survival benefit. This was consistent across both the 28-day mortality group (OR: 1.09, 95% CI: 0.74–1.60, Figure 5) and the 90-day mortality group (OR: 1.03, 95% CI: 0.87–1.23, Figure 6), suggesting no significant reduction in mortality at either time point.

**Figure 7:**
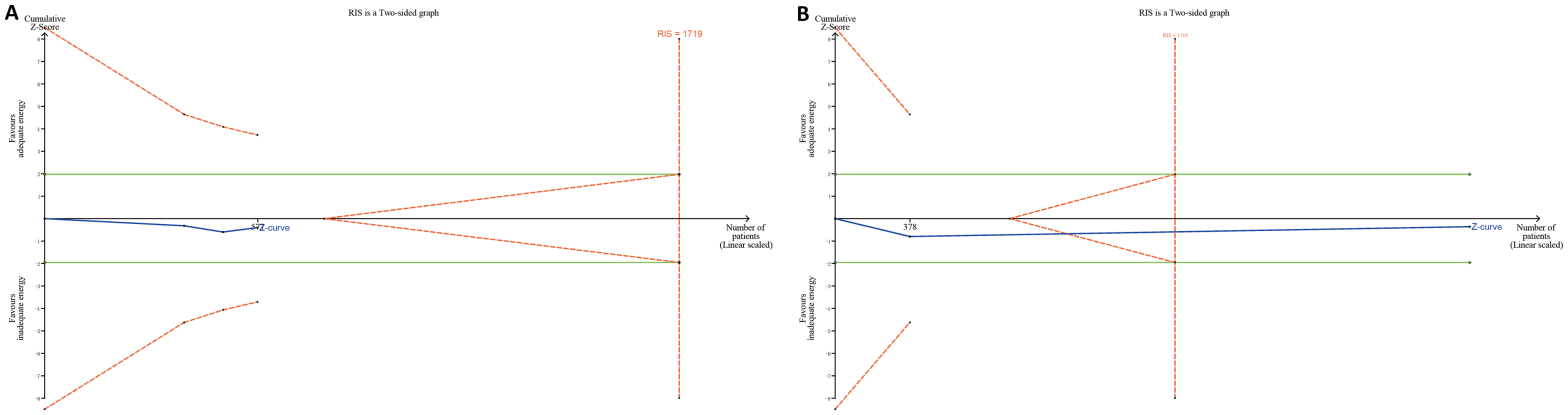
Trial sequential analyses in RCTs. **(A)** For the 28-day mortality group, the cumulative Z-curve did not cross either the conventional significance boundary (green dashed line) or the trial sequential monitoring boundary for benefit (red dashed line), nor did it reach the required information size (RIS) line. **(B)** For the 90-day mortality group, the Z-curve entered the futility area and crossed the RIS line, but it did not cross the conventional significance boundary.

TSA was performed separately for the 28-day and 90-day mortality subgroups, and no evidence was found to support a survival benefit from adequate energy intake. Notably, in the 90-day mortality group, the Z-curve crossed both the futility area and the required information size (RIS) line, suggesting that further increases in sample size are unlikely to demonstrate 90-day mortality benefit from adequate energy support in patients at high nutritional risk (Figure 7).

**Figure 8:**
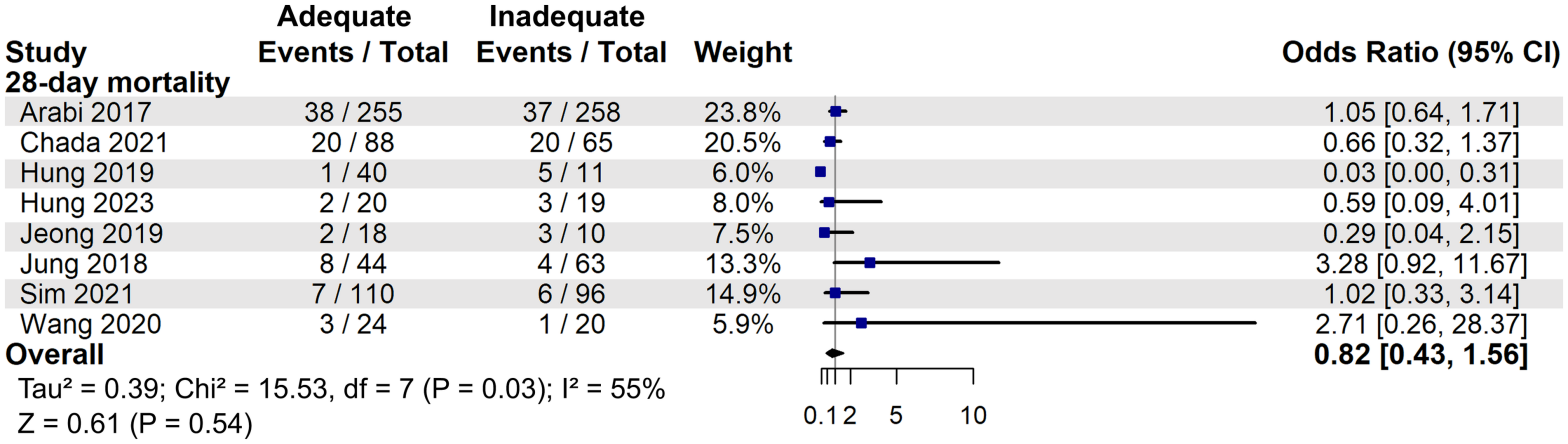
28-day mortality associated with energy adequacy in critically ill patients with low nutritional risk.

Although two studies (26, 28) suggested a potential mortality benefit of adequate energy support in low nutritional risk patients, our meta-analysis found no statistically significant difference compared to inadequate support in 28-day mortality (OR: 0.82, 95% CI: 0.43–1.56, *p* = 0.54, Figure 8). In the RCTs, adequate energy intake did not reduce 28-day mortality (OR: 1.05, 95% CI: 0.66–1.67, *p* = 0.84, Supplementary Figure S4) or 90-day mortality (OR: 1.08, 95% CI: 0.75–1.54, *p* = 0.68, Supplementary Figure S5).

### 3.5 Mortality associated with protein adequacy in critically ill patients

Among the 11 included studies, 4 investigated whether adequate protein intake is associated with a reduction in mortality rates among critically ill patients (22, 25, 27, 28). One suggested that adequate protein intake may reduce mortality in critically ill patients (28). However, meta-analysis demonstrated that adequate protein intake (≥1 g/kg/day) compared with relatively inadequate intake (<1 g/kg/day) did not significantly reduce mortality, regardless of whether the nutritional risk of critically ill patients is high (OR: 0.70, 95% CI: 0.38–1.30, *p* = 0.26, Supplementary Figure S6) or low (OR: 0.62, 95%CI: 0.09–4.13, *p* = 0.63, Supplementary Figure S7).

## 4 Discussion

This meta-analysis examined whether the mNUTRIC score is associated with mortality and whether it can serve as a tool for guiding nutritional support in critically ill patients. In patients with high nutritional risk, adequate energy support might contribute to reduced mortality. However, this association was not observed in the subgroup analysis limited to RCTs. Moreover, TSA analyses based on RCTs for both 28-day and 90-day mortality showed no trend toward a survival benefit from adequate energy support in high nutritional risk patients. In the 90-day analysis, the Z-curve crossed both futility boundary and RIS, suggesting that further RCTs are unlikely to demonstrate a significant survival benefit.

One possible explanation is that, in observational studies, patients with more severe illness may have inherently lower energy intake due to clinical limitations. As a result, higher mortality in the inadequate intake group may reflect underlying disease severity rather than the effect of insufficient nutrition itself. In contrast, the RCTs, through randomized allocation of nutrition strategies, minimized confounding and provided more reliable evidence. Therefore, the findings from the RCTs are considered more robust. Overall, while the mNUTRIC score remains a useful tool for risk stratification, its utility in guiding individualized energy provision strategies appears limited.

The results of our study were in agreement with those reported in large-scale RCTs, which have shown that early full nutritional support does not improve outcomes in critically ill patients (12, 33–37). For example, the EPaNIC trial found that harm was more related to the dose of nutrition rather than the route of administration. Similarly, the CALORIES and NUTRIREA-2 trials reported no mortality difference between enteral and parenteral nutrition. The NUTRIREA-3 trial, which allowed both routes, further showed worse outcomes with early high-dose feeding compared with permissive underfeeding.

Although the mNUTRIC score is widely recognized as a reliable tool for assessing risk of mortality (19–21), it does not appear to be a reliable indicator for guiding nutritional support strategies. Some studies, including the PermiT trial, suggest that it may not effectively identify patients at risk of malnutrition or those who are likely to benefit from nutritional support (23). Moreover, EFFORT trial similarly reached a comparable conclusion about mNUTRIC score (7). Therefore, nutritional support strategies may require greater emphasis on indicators that reflect the severity of illness rather than relying only on composite measures such as the mNUTRIC score based on our research.

Moreover, this study found a potential interaction between BMI and adequate energy intake: patients with BMI < 27 kg/m^2^ benefited from sufficient energy support, while those with BMI ≥ 27 kg/m^2^ showed no significant 28-day survival advantage. This finding is consistent with a previous study, which reported that increased calorie intake was associated with lower mortality in patients with BMI < 25 kg/m^2^ and ≥35 kg/m^2^, but conferred no benefit to those with BMI 25 kg/m^2^ to <35 kg/m^2^ (5). However, it should be noted that BMI has several limitations as a nutritional screening tool and requires careful consideration during application (38). Given the small number of available studies, this subgroup finding should be considered hypothesis-generating and requires prospective validation in future research.

Our study has several strengths and limitations. A notable strength is that we applied meta-regression techniques to explore potential sources of heterogeneity among the included studies, providing deeper insight into variability in treatment effects. Another strength of our study is the stratification of patients according to the mNUTRIC score, which allowed us to investigate the potential heterogeneity in the effect of early adequate nutritional support across different levels of nutritional risk in critically ill patients. Nonetheless, limitations include the following: (1) the limited number of high-quality studies, which may have impacted the stability of our conclusions; (2) the lack of consideration for the potential need to restrict energy intake within the first 72 h of the acute phase, as most included studies focused on feeding strategies over the first week; (3) the analysis of protein adequacy may be limited in statistical power, and therefore, the conclusions should be interpreted with caution; and (4) the conclusions may not reflect a limitation of the mNUTRIC score itself, but rather the discrepancy between identifying nutritional risk and ensuring the actual delivery of adequate nutritional support in clinical practice. Previous evidence suggests that mNUTRIC-based screening alone may not confer clinical benefit unless coupled with comprehensive multidisciplinary nutritional assessment and proactive interventions. Therefore, further high-quality studies are warranted to determine how best to integrate nutritional risk stratification with timely and adequate nutritional therapy to improve outcomes, so additional high quality studies are warranted to elucidate the appropriate timing, amount, and duration of early nutritional support in critically ill patients.

## 5 Conclusion

Our findings indicate that the mNUTRIC score possesses prognostic value for predicting mortality in critically ill patients; however, its role in guiding energy provision strategies remains limited.

## Data Availability

The original contributions presented in the study are included in the article/Supplementary material, further inquiries can be directed to the corresponding author.
