# Supplementary figures and images for: Impact of nutritional support on mortality among critically ill patients with different nutritional risks: a systematic review with meta-analysis
Source: Front Nutr. 2025 Nov 18;12:1667389. doi: 10.3389/fnut.2025.1667389 (PMC12669126; doi:10.3389/fnut.2025.1667389)

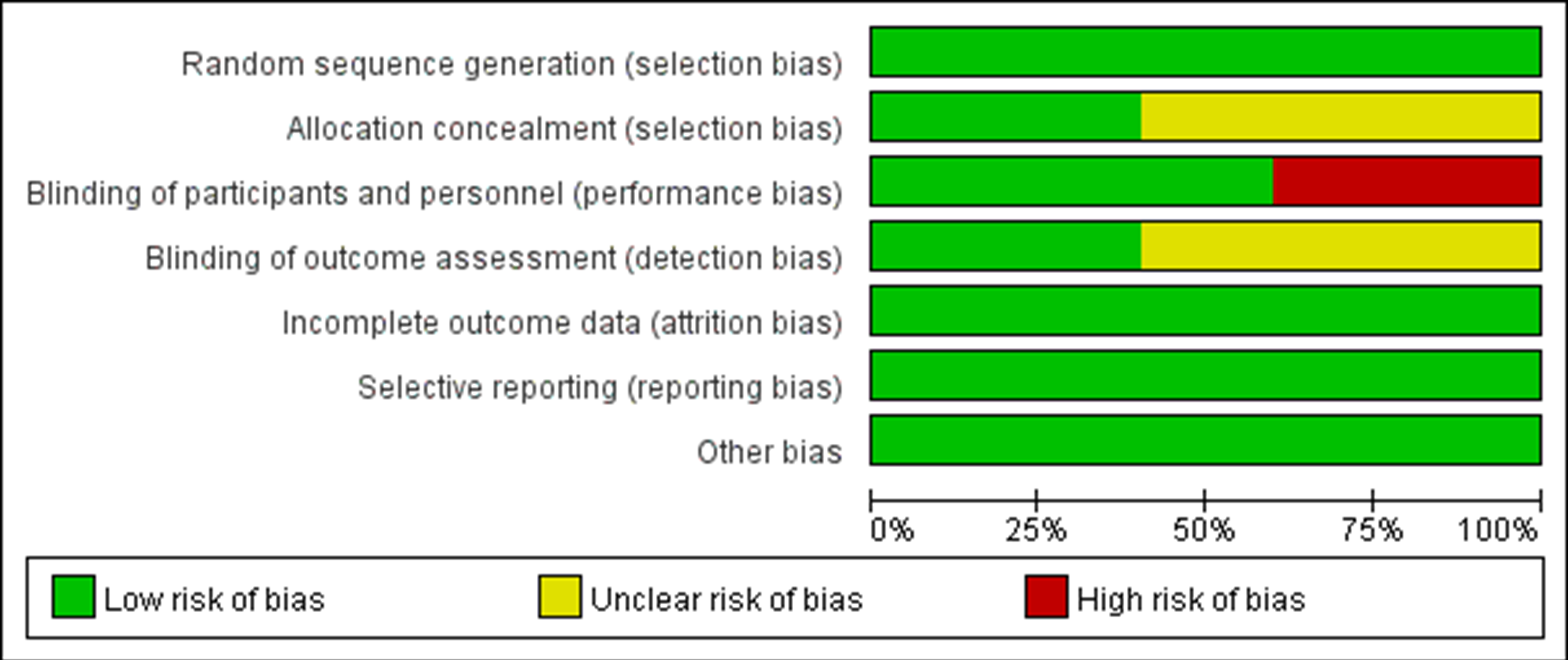

Supplement: Supplementary file 1 [file Image_1.TIF]

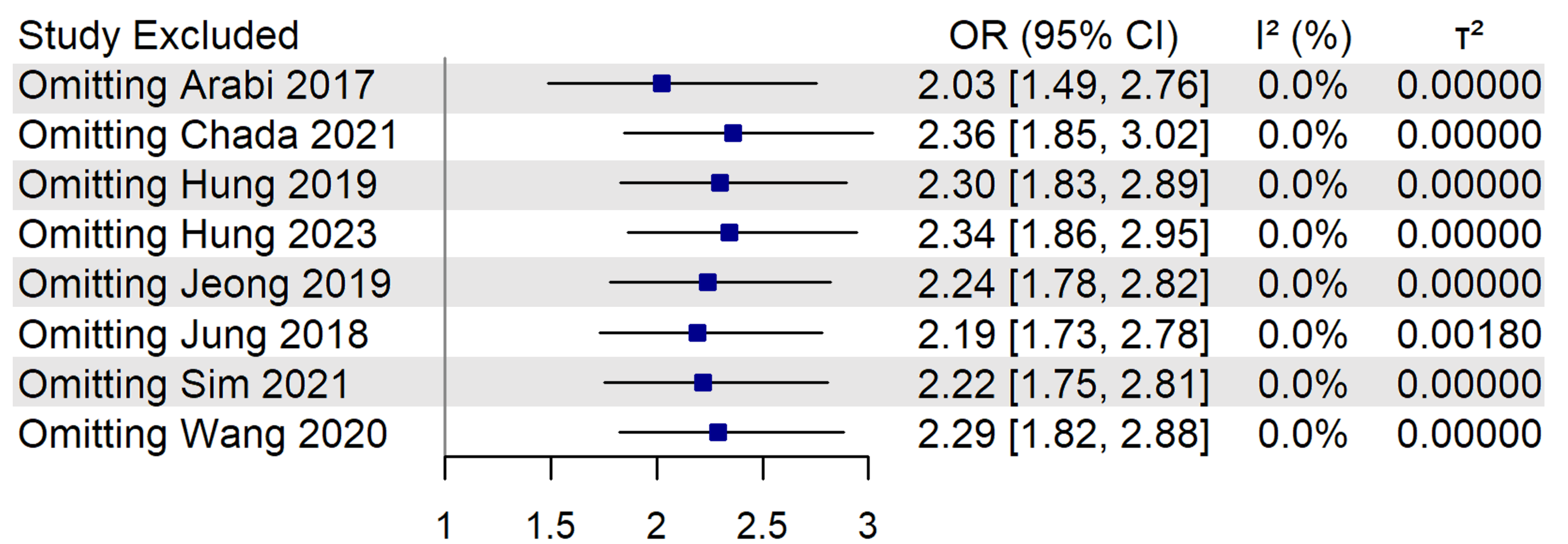

Supplement: Supplementary file 2 [file Image_2.TIF]

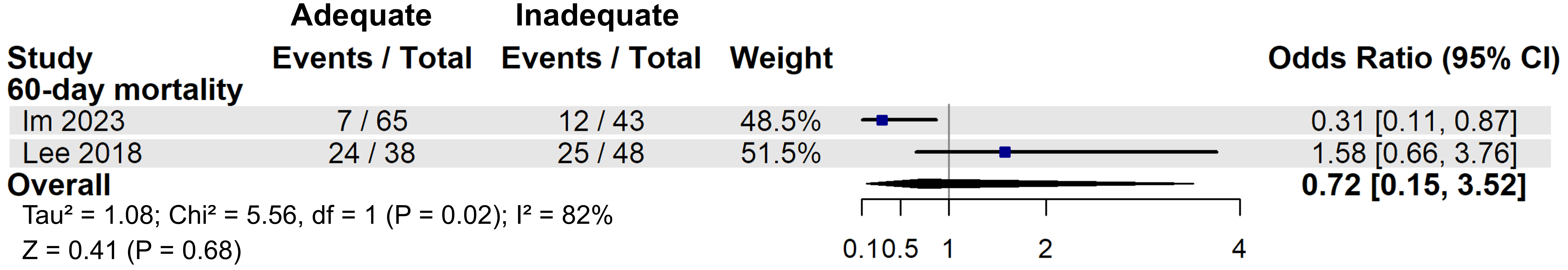

Supplement: Supplementary file 3 [file Image_3.TIF]

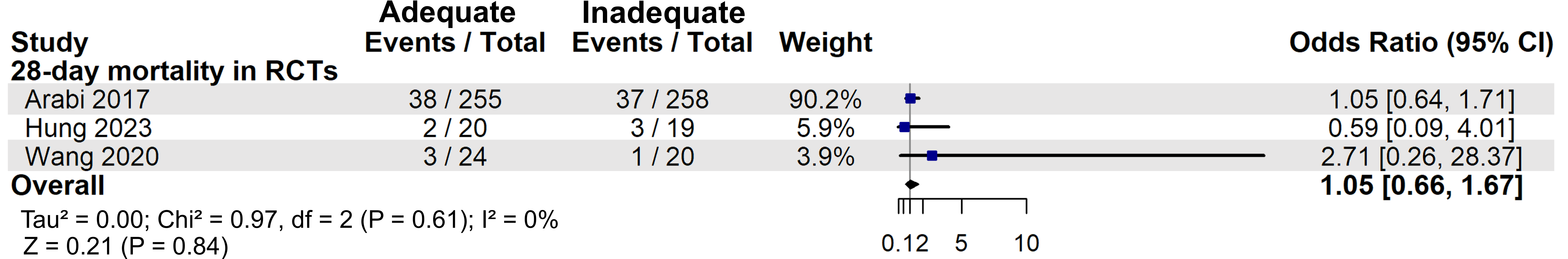

Supplement: Supplementary file 4 [file Image_4.TIF]

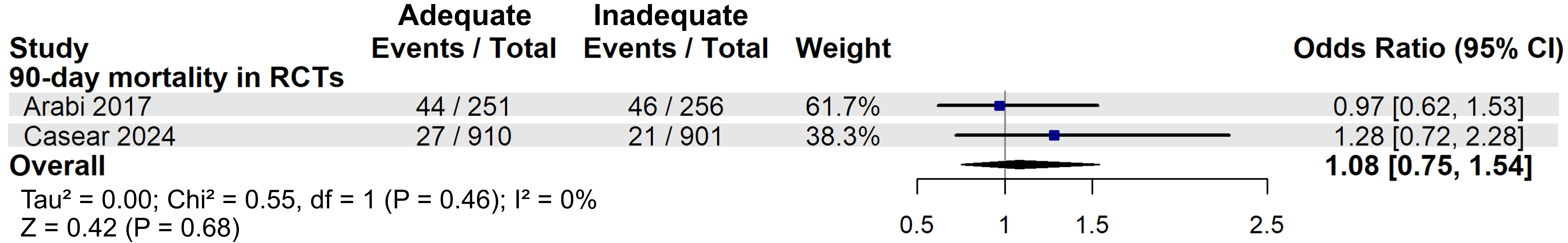

Supplement: Supplementary file 5 [file Image_5.TIF]

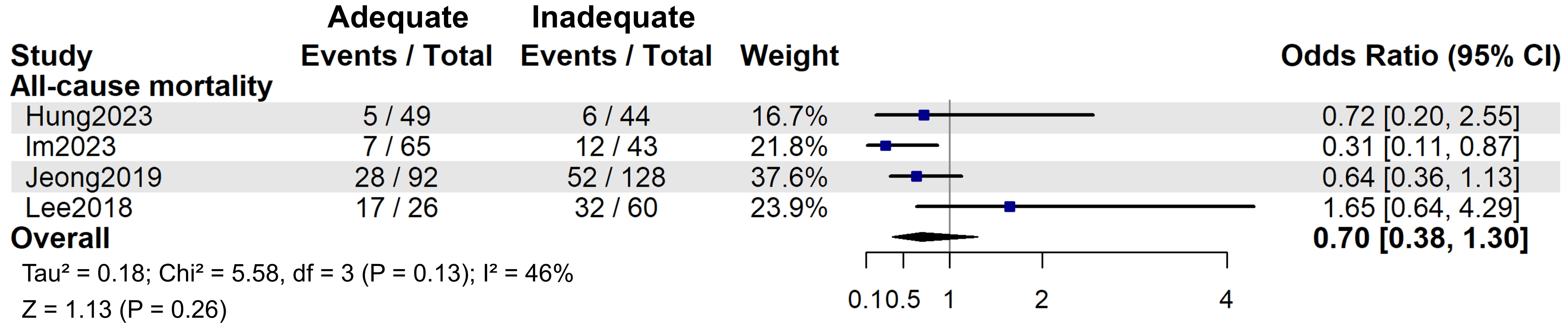

Supplement: Supplementary file 6 [file Image_6.TIF]

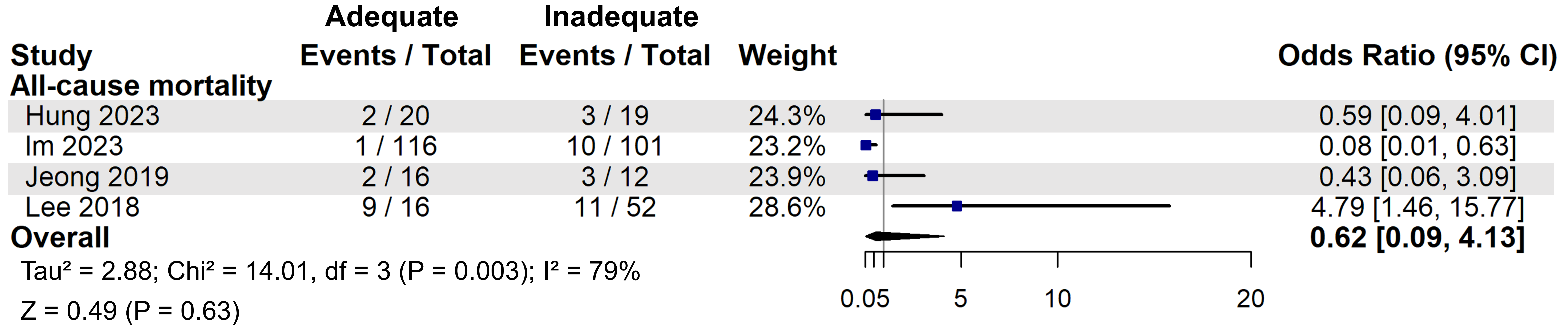

Supplement: Supplementary file 7 [file Image_7.TIF]
