# Supplementary material for: Impact of nutritional support on mortality among critically ill patients with different nutritional risks: a systematic review with meta-analysis
Source: Front Nutr. 2025 Nov 18;12:1667389. doi: 10.3389/fnut.2025.1667389 (PMC12669126; doi:10.3389/fnut.2025.1667389)
Supplement: Supplementary file 8 [file Table_1.DOCX]

**Table S1.** Quality and risk of bias assessment using the Newcastle-Ottawa Scale (NOS) for observational studies.

| **Study ID** | **Selection** | | | | **Comparability** | **Outcome** | | | **Total**  **(9*)** |
| --- | --- | --- | --- | --- | --- | --- | --- | --- | --- |
|  | Representativeness of the exposed cohort (*) | Selection of non-exposed cohort (*) | Ascertainment of exposure (*) | Demonstration that outcome of interest was not present at start of study (*) | Comparability of cohorts (**) | Assessment of outcome (*) | Follow up long enough for outcomes to occur (*) | Adequacy of follow up (*) |  |
| Chada 2021 | * |  | * | * |  | * | * | * | 6 |
| Hung 2019 | * |  | * | * |  | * | * |  | 5 |
| Jeong 2019 | * |  | * | * |  | * | * |  | 5 |
| Jung 2018 | * |  | * | * |  | * | * | * | 6 |
| Lee 2018 | * |  | * | * |  | * | * |  | 5 |
| Sim 2021 | * |  | * | * |  | * | * |  | 5 |
